# Histopathological and Transcriptional Changes in Silkworm Larval Gonads in Response to Chlorfenapyr Exposure

**DOI:** 10.3390/insects16060619

**Published:** 2025-06-11

**Authors:** Tao Li, Changxiong Hu, Zenghu Liu, Qiongyan Li, Yonghui Fan, Pengfei Liao, Min Liu, Weike Yang, Xingxing Li, Zhanpeng Dong

**Affiliations:** Sericulture and Apiculture Research Institute, Yunnan Academy of Agricultural Sciences, Mengzi 661101, China; hcxbear@126.com (C.H.); qmqc741@163.com (Z.L.); lqyyndl@163.com (Q.L.); fhyse@163.com (Y.F.); liaopengfei@yaas.org.cn (P.L.); liuumin@163.com (M.L.); wksun1985@163.com (W.Y.); lixxstarr@163.com (X.L.)

**Keywords:** *Bombyx mori*, chlorfenapyr, reproductive development, drug metabolism, hormone biosynthesis

## Abstract

Chlorfenapyr is a widely used insecticide known to harm non-target insects, but its effects on reproductive development in the silkworm (*Bombyx mori* L.) remain incompletely understood. In this study, we investigated the histopathological and transcriptional changes in the gonads (ovaries and testes) of newly molted fifth-instar silkworm larvae exposed to chlorfenapyr. Histopathological analysis revealed delayed gonadal development, a reduction in oogonia and oocytes in the ovaries, and decreased numbers of spermatocytes in the testes. Transcriptome analysis identified significant differentially expressed genes (DEGs), mainly enriched in pathways such as “Drug metabolism—cytochrome P450”, “Insect hormone biosynthesis”, and “Ribosome”. Key up-regulated genes included members of the cytochrome P450 family (*CYP6B5*, *CYP9f2*, *CYP6B6*), glutathione S-transferases (*GSTT1*, *GST1*), and juvenile hormone-related enzymes (*JHAMT*, *JHEH*), indicating active detoxification and hormonal regulation responses. Several transcription factor families, particularly C2H2, HB-other, and TRAF, exhibited altered expression, suggesting roles in stress adaptation. Protein–protein interaction (PPI) network analysis identified hub genes such as *EcR*, *Kr-h1*, and various ribosomal proteins, highlighting their potential involvement in reproductive development. Quantitative PCR (qPCR) validated the transcriptomic data, confirming the reliability of the results. Overall, these findings enhance our understanding of chlorfenapyr’s impact on silkworm reproductive development and the underlying molecular mechanisms, providing valuable insights for sustainable pest management and ecological risk assessment of insecticides.

## Simple Summary

*Bombyx mori* is not only an important agricultural economic insect but also a representative lepidopteran model insect. Due to long-term indoor breeding and domestication, silkworms are highly sensitive to many chemical pesticides, and they serve as a research model for evaluating the reproductive development of pesticides on organisms. This study investigates the histopathology and transcriptome of gonads (ovaries and testes) of silkworms following exposure to chlorfenapyr. After exposure for 72 h and 96 h, chlorfenapyr caused abnormal development in larval silkworm gonads. At a transcriptome level, DEGs were primarily associated with drug metabolism—cytochrome P450, drug metabolism—other enzymes, and insect hormone biosynthesis. A regulatory network is constructed to identify key responsive proteins. These findings provide new insights into the molecular mechanism by which chlorfenapyr affects reproductive development.

## 1. Introduction

*Bombyx mori* is an agriculturally important insect that plays a central role in the production of silk [1,2]. The growth and development of silkworms depend on the availability of healthy mulberry leaves, their primary food source, but mulberry trees are susceptible to pests [3]. To ensure the healthy growth of silkworms, farmers must implement effective control measures, including the use of pesticides to safeguard mulberry trees from pests. These insecticides may directly affect silkworm growth and cocoon production [4,5,6].

Chlorfenapyr, a pyrrole insecticide, is highly efficient and minimally toxic, leading to its widespread use for controlling agricultural pests [7]. It acts by inhibiting ATP synthesis during mitochondrial oxidative phosphorylation, disrupting energy metabolism [8]. Chlorfenapyr significantly inhibits the growth of S9 cells and simultaneously reduces ATP concentration [9]. Research has primarily focused on the sublethal effects of chlorfenapyr on growth, development, and physiological responses in insects such as *Spodoptera frugiperda* [10], *Plutella xylostella* [11], and *Bradysia odoriphaga* [12]. However, its impact on insect reproductive development remains poorly understood. It exhibits cytotoxic effects on Asian palm weevil *Rhynchophorus ferrugineus* midgut cells (RW-1) [13]. Moreover, chlorfenapyr can also cross the blood–brain barrier, suggesting a potential association with brain lesions in mice [14]. Detoxification mechanisms used by silkworm after exposure to chlorfenapyr have been reported [15]. Our previous investigation revealed that chlorfenapyr stress can impact the economic traits and fecundity of silkworm [16]. However, the molecular mechanism by which chlorfenapyr affects reproductive development in silkworms remains poorly understood.

We investigate the histopathology and transcriptome of gonadal (ovarian and testicular) tissues of silkworm following exposure to chlorfenapyr. Related gene expression changes and the regulatory network are analyzed to describe how chlorfenapyr affects the silkworm reproductive system. The results can be used to establish more sustainable use of chlorfenapyr for pest control and improve our understanding of its potential toxicity to non-target species.

## 2. Materials and Methods

### 2.1. Silkworm Samples and Chlorfenapyr Stress

The *P50* silkworm strain and mulberry leaves used in this study were obtained from our laboratory. Silkworms were reared under standard conditions (26 °C, 70 ± 5% relative humidity). At the newly molted fifth-instar stage, larvae were divided into two groups: the treatment group was fed mulberry leaves treated with 100 mg/L of chlorfenapyr [16], while the control group received untreated leaves. Larvae from both groups were collected after 72 h or 96 h of feeding, and their gonads (ovaries and testes) were carefully dissected. These tissues were subjected to histopathological and transcriptomic analyses. The samples were sorted into eight groups, each consisting of tissues from twelve ovaries or testes (from six larvae). Treatment groups were designated as OT-72h, OT-96h, TT-72h, and TT-96h, while control groups were named OC-72h, OC-96h, TC-72h, and TC-96h. All groups were processed in triplicate to ensure reproducibility.

### 2.2. Ovary and Testis Histopathology

Ovarian and testicular tissues were fixed in 4% paraformaldehyde for 24 h. After fixation, the tissues were dehydrated through a graded ethanol series, cleared with xylene, embedded in paraffin, and sectioned into 5 µm slices. Sections were deparaffinized, stained with hematoxylin and eosin (HE), sealed with neutral gum, and examined under a light microscope (Nikon Eclipse CI, Tokkyo, Japan).

### 2.3. Transcriptome Sequencing (RNA-Seq) and Analysis

Total RNA was extracted from treated and control tissues using TRIzol reagent (Invitrogen, Carlsbad, CA, USA) according to the manufacturer’s protocol. RNA concentration and purity were assessed using a Nanodrop 2000 spectrophotometer (Thermo Scientific, Waltham, MA, USA), and integrity was evaluated using an Agilent RNA 6000 Nano Kit and a Bioanalyzer 2100 system (Agilent Technologies, Santa Clara, CA, USA). A total of 24 sequencing libraries were constructed.

mRNA was isolated using Oligo(dT) magnetic beads, fragmented (~300 bp), and used for first-strand cDNA synthesis with random primers. Second-strand synthesis, end repair, adaptor ligation, and PCR amplification were subsequently performed to generate the final libraries.

Sequencing was carried out on an Illumina NovaSeq 6000 platform. Raw reads were quality-filtered using FASTP [17], and clean reads were obtained by removing adapter sequences and low-quality reads. HISAT2 [18] was used for sequence alignment, and StringTie [19] was employed to assemble transcripts and identify novel genes.

### 2.4. Differential Gene Expression and Gene Enrichment Analyses

DESeq2 was used to identify differentially expressed genes (DEGs) based on expression levels, applying thresholds of |log_2_(fold change)| ≥ 0.263 and Q-value (adjusted *p*-value) ≤ 0.05 [20]. Functional enrichment of DEGs was assessed via KEGG pathway analysis using KOBAS [21]. GO and KEGG enrichment analyses were performed using the *phyper* function in R, with *p* ≤ 0.05 considered statistically significant.

### 2.5. Creating a Protein–Protein Interaction (PPI) Network

The Search Tool for the Retrieval of Interacting Genes/Proteins (STRING) database (URL accessed on 12 October 2024, https://string-db.org/) was used to investigate protein–protein interactions (PPIs) among predicted and known proteins. STRING is a comprehensive database covering 2,031 species and providing information on 13.8 million interactions involving 9.6 million proteins. Analyzing the interaction network of proteins helps identify key regulatory genes. The PPI network of differentially expressed proteins (DEPs) was constructed using STRING (v.9.1) [22], and the resulting network was visualized using Cytoscape (v.2.8) [23].

### 2.6. Quantitative Real-Time Polymerase Chain Reaction (qPCR) Analysis

Total RNA was extracted using TRIzol reagent (Takara, Dalian, China). First-strand cDNA synthesis was conducted using the HiScript III RT SuperMix for qPCR (Vazyme, Nanjing, China), following the manufacturer’s instructions. Primers for target genes were designed using Primer-BLAST (URL accessed on 26 October 2024, https://www.ncbi.nlm.nih.gov/tools/primer-blast/index.cgi?LINK_LOC=BlastHome) and synthesized by Qingke Biotechnology (Beijing, China). The major primer sequences used for qPCR are listed in Table S1. Candidate genes identified from RNA-seq analysis were validated via qPCR using NovoStart SYBR qPCR SuperMix Plus (Novoprotein, Shanghai, China). qPCR was performed using an Applied Biosystems StepOne Plus system (Applied Biosystems, Foster, CA, USA), following a previously described protocol [24] with minor modifications. The thermocycling conditions were as follows: initial denaturation at 95 °C for 10 min, followed by 40 cycles of 95 °C for 15 s and 60 °C for 30 s, and a final dissociation stage. Each sample was analyzed in triplicate. Relative gene expression levels were calculated using the 2^−ΔΔCT^ method [25] with normalization to the reference gene *BmGAPDH*. Statistical analysis was performed using one-way ANOVA to compare gene expression levels between treatment and control groups, followed by Tukey’s multiple comparison test to assess significance among all sample groups (*p* ≤ 0.05).

## 3. Results

### 3.1. Structure of Larval Silkworm Gonad After Chlorfenapyr Stress

**Figure 1:**
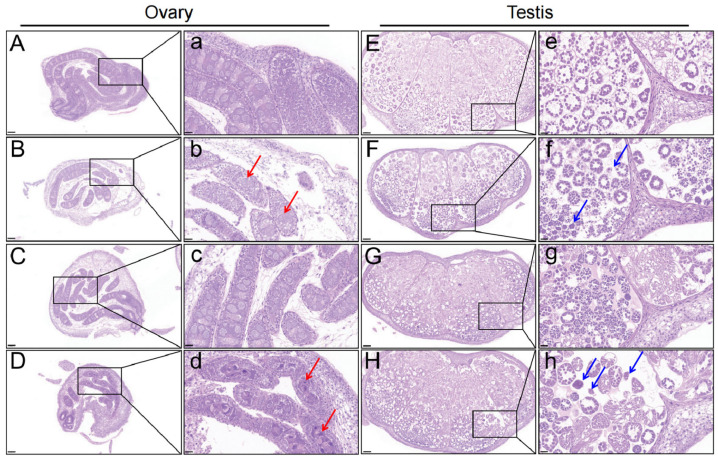
Histological structure of ovary and testis tissues in silkworm larvae following exposure to chlorfenapyr (HE staining). (**A**–**D**) represent the OC-72h, OT-72h, OC-96h, and OT-96h groups (10×), respectively. (**E**–**H**) represent the TC-72h, TT-72h, TC-96h, and TT-96h groups (10×), respectively. The lowercase letters “**a**–**h**” represent magnifications of the screenshots (40×). The red arrows indicate abnormal oocyte development, including reduced numbers. The blue arrows indicate spermatocytes exhibiting developmental delay and tightly aggregated nuclei.

After 72 h and 96 h, ovarian sections in controls (OC-72h and OC-96h) were normal, with evenly distributed follicles, primary oocytes, and interstitial tissues (Figure 1A,a,C,c). However, in the OT-72h and OT-96h groups, ovarian size decreased, development was delayed, and the number of oogonia and oocytes decreased, especially after 96 h (Figure 1B,b,D,d). Testis sections in control silkworms (TC-72h and TC-96h) remained normal, with normal spermatogonia and evenly distributed chromatin (Figure 1E,e,G,g). In treatment silkworms (TT-72h and TT-96h), testis development was significantly delayed, and spermatocytes showed tightly aggregated nuclei and reduced numbers (Figure 1F,f,H,h).

### 3.2. Analysis of Differentially Expressed Genes Between Treatments

**Figure 2:**
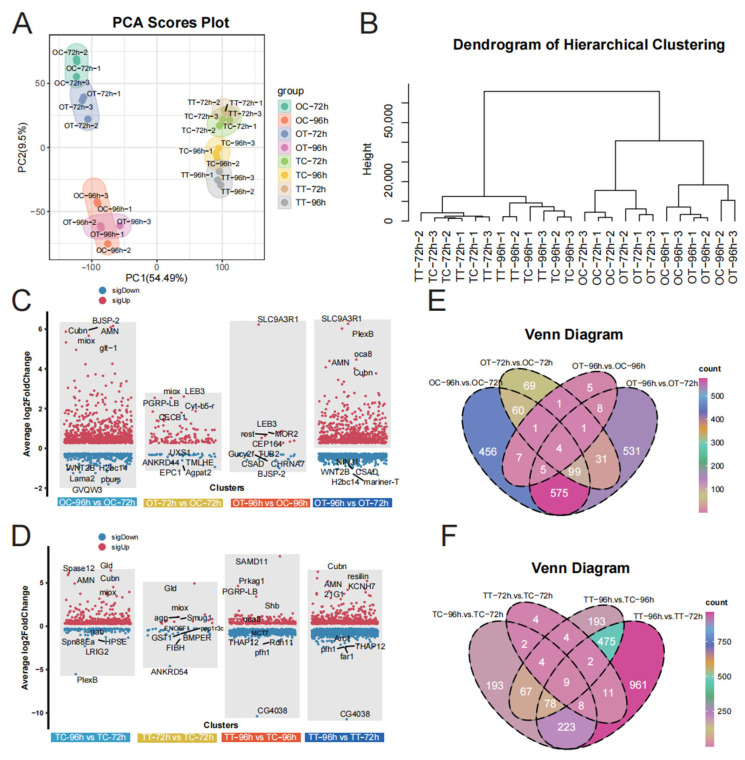
Transcriptome sequencing results. (**A**) PCA of transcriptome data. (**B**) Hierarchical cluster analysis (HCA) of transcriptome data. (**C**) The volcano plot shows the differentially expressed genes (DEGs) in ovary. (**D**) The volcano plot shows the differentially expressed genes (DEGs) in testis. (**E**) Venn diagrams for transcriptome analysis of the DEGs detected between different groups in ovary. (**F**) Venn diagrams for transcriptome analysis of the DEGs detected between different groups in testeis.

The quality of the raw transcriptome data was initially assessed, and statistical analysis of the RNA sequencing data is presented in Table S2. Principal component analysis (PCA) revealed distinct differences between ovary and testis tissues after 72 h and 96 h of treatment (Figure 2A), indicating that treatment duration differentially affected the development of these tissues. Hierarchical clustering analysis showed high similarity among biological replicates and clear distinctions between the TT-72h and TT-96h groups, as well as between the OT-72h and OT-96h groups (Figure 2B). These results confirm the stability and reproducibility of the data, ensuring the reliability of the findings.

To explore gene expression patterns in silkworm reproductive tissues after chlorfenapyr exposure, we compared the number of DEGs across treatment groups. More DEGs were detected in the OC-96h vs. OC-72h and OT-96h vs. OT-72h comparisons (Figure 2C, Table S3). Compared with OC-72h treatment, we identified 1207 DEGs in the OC-96h treatment and 171 in the OT-72h treatment (Figure 2C, Table S2). A total of 1254 DEGs were identified in the OT-96h vs. OT-72h comparison (Figure 2C, Table S3). Additionally, in testis tissues, we found 584 DEGs in the TC-96h vs. TC-72h comparison, 44 in the TT-72h vs. TC-72h comparison, 832 in the TT-96h vs. TC-96h comparison, and 1767 in the TT-96h vs. TT-72h comparison (Figure 2D, Table S4). Among them, only four and nine shared genes were identified in ovary (Figure 2E) and testis (Figure 2F) tissues, respectively. These results indicate that most genes were specifically expressed compared to their respective control groups.

### 3.3. KEGG Pathway Enrichment Analysis of DEGs

**Figure 3:**
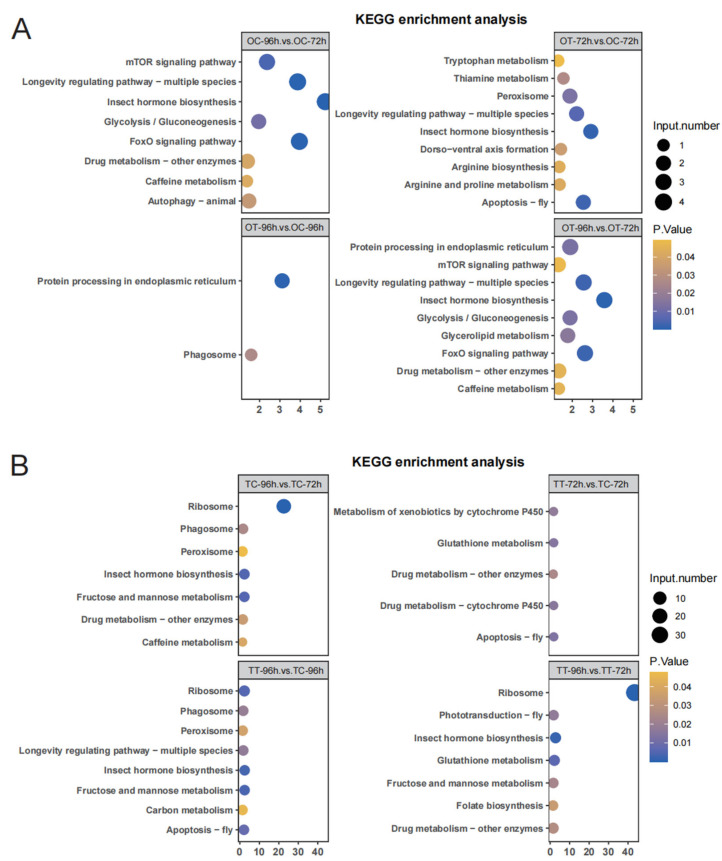
The significant Kyoto Encyclopedia of Genes and Genomes (KEGG) transcriptome annotation results of DEGs. (**A**) KEGG pathway analysis of DEGs in the four comparison groups of ovary tissues. (**B**) KEGG pathway analysis of DEGs in the four comparison groups of testis tissues. Pathways with the *p*-values < 0.05 were used to draw the map.

To explore the functions of genes potentially related to the silkworm’s response to chlorfenapyr, we performed KEGG enrichment analysis on DEGs in each treatment. DEGs in the OT-96h and OC-96h treatments were mainly involved in the “Protein processing in endoplasmic reticulum” and “Phagosome” pathways (Figure 3A). In the OT-72h vs. OC-72h comparison, DEGs were mainly enriched in “Insect hormone biosynthesis”, “Apoptosis—fly”, and “Longevity regulating pathway—multiple species” (Figure 3A). Those in the OT-96h vs. OT-72h comparison were significantly enriched in “Insect hormone biosynthesis”, “Longevity regulating pathway—multiple species”, “FoxO signaling pathway”, and “Drug metabolism—other enzymes” (Figure 3A). In testis tissues, for the TT-96h vs. TC-96h comparison, significant enrichment occurred in “Ribosome”, “Insect hormone biosynthesis”, “Fructose and mannose metabolism”, and “Apoptosis—fly” (Figure 3B). DEGs in the TT-72h vs. TC-72h comparison were most enriched in “Metabolism of xenobiotics by cytochrome P450”, “Drug metabolism—cytochrome P450”, and “Apoptosis—fly” (Figure 3B). In the TT-96h vs. TT-72h comparison, “Insect hormone biosynthesis” and “Glutathione metabolism” were significantly enriched (Figure 3B). These KEGG results indicate that the DEGs in reproductive tissues in different comparisons were mainly associated with “Drug metabolism—cytochrome P450”, “Drug metabolism—other enzymes”, and “Insect hormone biosynthesis” (Figure 3A,B).

### 3.4. Transcriptional Changes of P450, Glutathione S-Transferase (GST), and Insect Hormones

**Figure 4:**
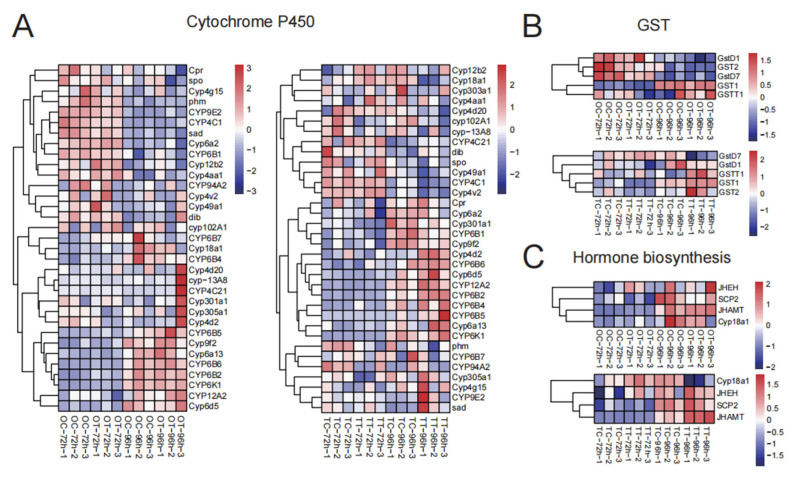
Hierarchical cluster analysis of important genes in different groups of silkworm gonads. (**A**) Cytochrome P450; (**B**) GST; (**C**) hormone biosynthesis.

Because “Drug metabolism” and “Insect hormone biosynthesis” were significantly enriched, chlorfenapyr affects drug metabolism and hormone regulation processes in larval silkworm reproductive tissues. Cytochrome P450 and glutathione S-transferase (GST) play important roles in the detoxification process. In ovary tissues, we found that *Cyp12b2* and *Cyp4aa1* were up-regulated in the OT-72h treatment compared with the OC-72h treatment (Figure 4A). Additionally, *CYP6B5*, *Cyp9f2*, *CYP6B6*, *CYP12A2*, and *Cyp6d5* were more highly expressed in the OT-96h treatment than in the OC-96h treatment (Figure 4A). In testis tissues, *Cyp4aa1* and *Cyp4v2* were up-regulated in the TT-72h treatment, while *Cyp4d2*, *CYP6B6*, *Cyp6d5*, *CYP12A2*, *CYP6B2*, and *CYP6B4* were more highly expressed in the TT-96h treatment compared with the TC-96h treatment (Figure 4A). For GST, *GSTT1* was significantly up-regulated in the OT-96h treatment, and both *GSTT1* and *GST1* were up-regulated in the TT-96h treatment (Figure 4B). Changes in the “Insect hormone biosynthesis” pathway may reflect adaptive adjustments of insects to different physiological states or environmental conditions. Notably, *JHAMT* was significantly up-regulated in the OT-96h treatment, while *JHEH* and *JHAMT* were up-regulated in the TT-96h treatment (Figure 4C).

### 3.5. Prediction of Transcription Factors and Their Expression Patterns

**Figure 5:**
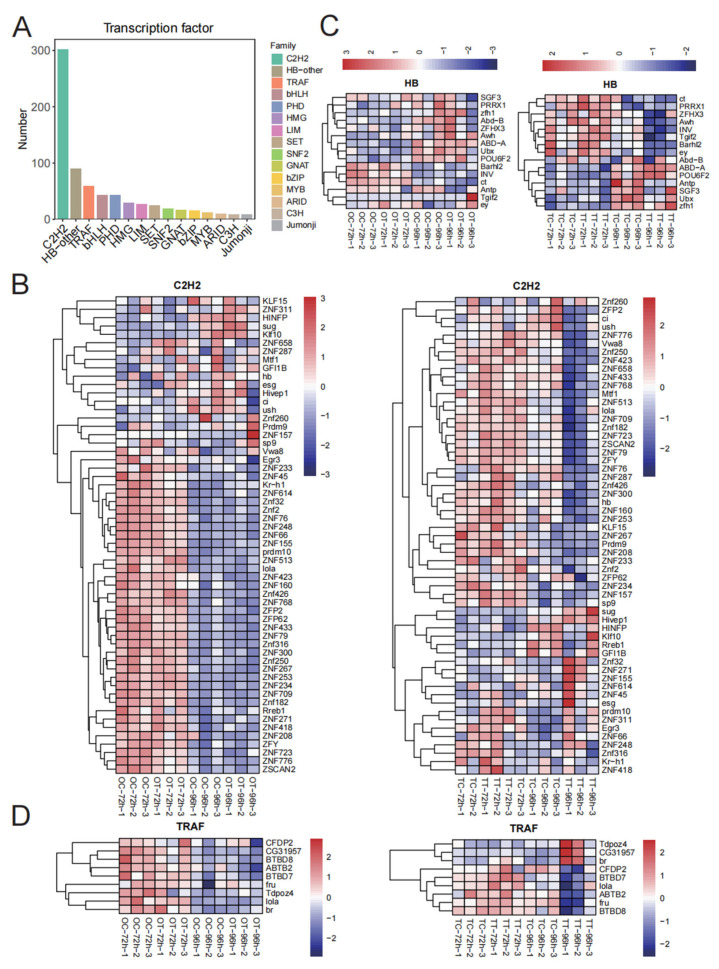
Expression of transcription factors. (**A**) Number of transcription factors; (**B**) transcription factors’ expression patterns of C2H2; (**C**) transcription factors’ expression patterns of HB; (**D**) transcription factors’ expression patterns of TRAF.

We identified 775 transcription factors, which were clustered into 39 families (Table S5). The top 15 of these were selected for visualization; the C2H2 family had the most representatives, followed by HB-other, and TRAF families (Figure 5A). In the OT-96h treatment, most C2H2 transcription factors were down-regulated compared with the OT-72h treatment, and a similar trend was observed in the TT-96h treatment in testis tissues (Figure 5B). For HB transcription factors, we found that ct, PRRX1, ZFHX3, Awh, INV, Tgif2, Barhl2, and ey were up-regulated in the TT-72h treatment compared with the TC-72h treatment (Figure 5C). Additionally, expression of ABD-A in the TT-96h treatment was up-regulated compared with the other three groups (Figure 5C). Most TRAF transcription factors were down-regulated in the OT-72h treatment compared with the OC-72h treatment. In the TT-96h treatment, CFDP2, BTBD7, lola, ABTB2, fru, and BTBD8 were down-regulated compared with other groups (Figure 5D).

### 3.6. Analysis of PPI Network

**Figure 6:**
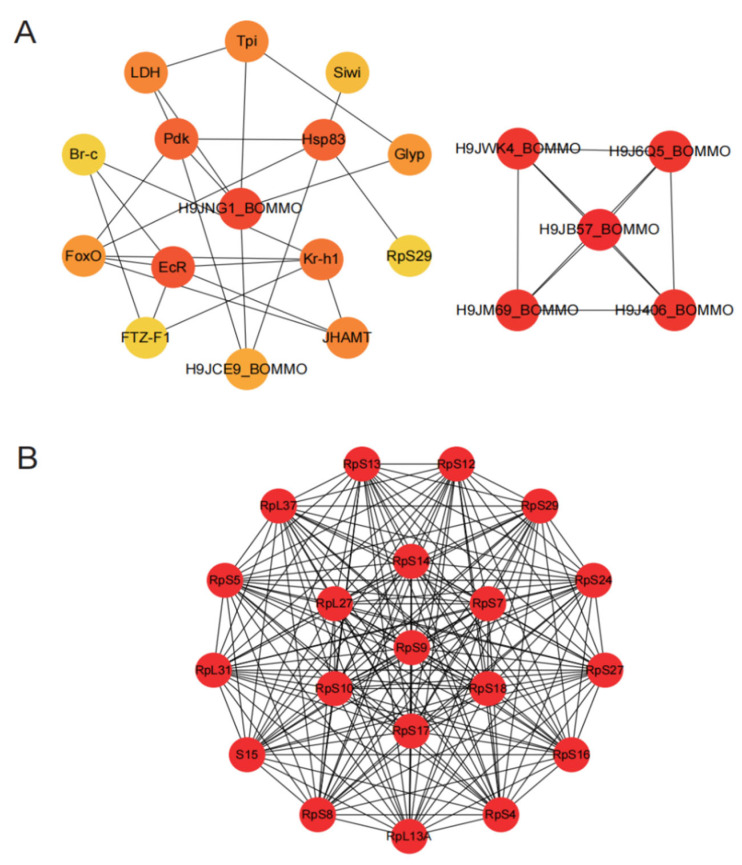
Construction of PPI network and screening of hub genes. (**A**) PPI results of DEGs in ovary tissues, and the top 20 DEGs were ranked by the MCC algorithm. (**B**) PPI results of DEGs in testis tissues, and the top 20 DEGs were ranked by the MCC algorithm. The red color indicates nodes with high MCC scores, and yellow indicates nodes with low MCC scores.

We used the PPI network to identify target genes in reproductive tissues under chlorfenapyr stress. The top 20 genes (e.g., H9JNG1_BOMMO, *EcR*, *Pdk*, *Hsp83*, *Kr-h1*, *FoxO*, *JHAMT*, *FTZ-F1*, and *Siwi*) were identified using a maximal clique centrality ranking method in ovary tissues (Figure 6A). These genes play important roles in apoptosis, carbohydrate metabolism (including starch and sucrose metabolism, fructose and mannose metabolism, and glycolysis/gluconeogenesis), insect hormone biosynthesis, and protein processing in the endoplasmic reticulum. In testis tissues, using this same method, the top 20 genes included *RpS9*, *RpS18*, *RpS14*, *RpL27*, *RpS10*, *RpS17*, *RpS7*, *RpS4*, *RpS16*, *RpS27*, *RpS24*, *RpS29*, *RpS12*, *RpS13*, *RpS5*, *S15*, *RpS8*, *RpL13A*, *RpL37*, and *RpL31* (Figure 6B). Based on KEGG analysis, these hub genes were primarily enriched in the ribosome pathway.

Chlorfenapyr profoundly affects the silkworm reproductive system, particularly in protein synthesis and cellular metabolism. Enrichment of ribosomal protein genes indicates that chlorfenapyr may disrupt normal cellular function by inhibiting protein synthesis, potentially leading to abnormal gonad development. Furthermore, key genes identified in the ovary (e.g., *EcR*, *Kr-h1*) reveal the significant role of insect hormones in reproductive processes. Interference with these hormones may represent an important mechanism by which chlorfenapyr affects reproductive development.

### 3.7. Validation of Gene Expression Patterns After Exposure to Chlorfenapyr

**Figure 7:**
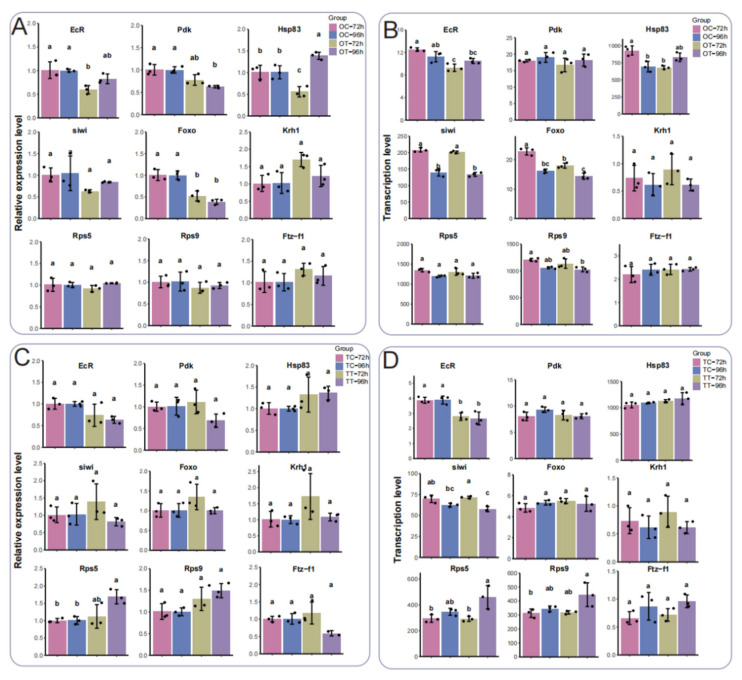
Comparison of RNA-seq and qPCR analyses for the selected DEGs. (**A**) Relative expression profiling of DEGs in the ovary by qPCR analysis. (**B**) Transcription level of DEGs in the ovary by RNA-seq. (**C**) Relative expression profiling of DEGs in the testis by qPCR analysis. (**D**) Transcription level of DEGs in the testis by RNA-seq. Different lowercase letters represent significant differences (*p* ≤ 0.05).

To verify the reliability of transcriptome data, we selected several DEGs for qPCR analysis. *EcR* and *Hsp83* had the lowest expressions in the OT-72h treatment relative to other treatments (Figure 7A), consistent with transcriptome analysis (Figure 7B). The qPCR results indicate that the expression trend of *EcR* matched the sequencing data, with no significant differences in *Krh1*, *Rsp5*, and *Ftz-f1* (Figure 7A,B). In testis tissues, expression of *Rps5* was up-regulated in the TT-96h treatment, and there were no significant differences in *Pdk*, *Hsp83*, *Foxo*, *Krh1*, and *Ftz-f1* in both qPCR and sequencing data (Figure 7C,D). The transcription levels of *EcR* in both the TT-72h and TT-96h treatments were down-regulated compared with the TC-72h and TC-96h treatments, and a similar tendency was apparent in the qPCR results (Figure 7C,D). In qPCR and sequencing data for the TT-72h treatment, *siwi* had the highest expression, whereas in the TT-96h treatment, *Rps5* and *Rps9* were most highly expressed (Figure 7C,D). These analyses reveal RNA sequencing data to be consistent and reliable, validating the gene expression trends observed through qPCR.

## 4. Discussion

Chlorfenapyr is a widely used insecticide that exhibits toxicity to a broad range of insects [26]. Its exposure induces significant sublethal effects, including impaired growth, altered enzyme activity, and reduced nutrient reserves in test insects [10,11,12]. In this study, we observed anatomical changes in silkworm gonads (ovaries and testes) following chlorfenapyr exposure. To further investigate the effects of chlorfenapyr on these reproductive tissues, transcriptome sequencing was conducted after silkworms were fed chlorfenapyr-treated mulberry leaves for 72 h and 96 h. Chlorfenapyr influenced the transcriptomic profiles of both ovaries and testes, with the highest number of DEGs identified between OT-96h and OT-72h, and TT-96h and TT-72h. These DEGs were primarily enriched in pathways related to drug metabolism—cytochrome P450 and insect hormone biosynthesis.

We posit that chlorfenapyr may lead to maldevelopment of the silkworm reproductive system by interfering with key biological processes. Specifically, in the OT-96h treatment, the expression of cytochrome P450 family genes such as *CYP6B5*, *CYP9f2*, *CYP6B6*, *CYP12A2*, and *CYP6d5* was significantly up-regulated compared with those in the control treatment. In the TT-96h treatment, the expression levels of *CYP4d2*, *CYP6B6*, *CYP6d5*, *CYP12A2*, *CYP6B2*, *CYP6B4*, *CYP6B5*, and *CYP6A13* were higher than those in the TC-96h treatment. Cytochrome P450 is involved in detoxification in insects [27]. Many members of the CYP9 family are involved in detoxification pathways related to insecticide resistance [28]. The CYP4 and CYP6 genes are also involved in the metabolism and detoxification of exogenous compounds in some insects. We report the up-regulation of CYP4 and CYP6 genes, indicating that silkworms primarily rely on these P450 family genes for detoxification when consuming mulberry leaves containing chlorfenapyr. Significant changes in cytochrome P450 family genes may suggest that silkworms experience a considerable metabolic burden when detoxifying, which could further damage their reproductive system.

GSTs are a multifunctional protein superfamily that plays important roles in the detoxification of insecticides and exogenous compounds in insects [29]. We report that *GSTT1* and *GST1* (*GSTM1*) are up-regulated in both the OT-96h and TT-96h treatments. *GSTT1* gene polymorphism may affect an individual’s ability to metabolize drugs and toxins [30]. *GSTM1* and *GSTT1* are cell detoxification enzymes that are involved in converting free radicals derived from reduced oxygen (known to harm DNA) into oxidized molecules that are not harmful to DNA [31]. Cells deficient in either *GSTM1* or *GSTT1* have impaired cellular detoxification, exposing them to free radicals derived from the environment, unhealthy nutrition, or some drugs. Therefore, up-regulation of *GSTM1* and *GSTT1* indicates that the silkworm has responded positively to chlorfenapyr exposure.

We report significant up-regulation of the *JHAMT* gene (associated with insect hormone synthesis) in the OT-96h treatment and observe upward trends for both *JHEH* and *JHAMT* in the TT-96h treatment. Juvenile hormone (JH) acid methyltransferase (*JHAMT*) is a rate-limiting enzyme that converts JH acids or their inactive precursor into active JH in the final step of biosynthesis in insects [32,33]. Changes in *JHAMT* expression directly affect the synthesis of JH acids, thereby influencing insect growth, development, molting, metamorphosis, and reproduction [34,35]. Juvenile hormone epoxide hydrolase (*JHEH*) participates in degrading JH acids, thus also regulating JH concentration and influencing insect developmental rhythms [36,37]. If the up-regulation of *JHAMT* and *JHEH* that we report is related to chlorfenapyr stress, it may represent an adaptive response to specific environmental pressures or growth demands. This further demonstrates the impact of chlorfenapyr on JH metabolism and multiple physiological developmental processes, including growth, development, and reproduction in silkworms.

The transcription factors identified from DEGs mainly clustered into 15 families (Figure 5A). Of these, the top three were C2H2, HB-other, and TRAF, of which most were significantly down-regulated in the TT-96h treatment. C2H2 transcription factors can regulate the expression of related genes in response to environmental stress (e.g., temperature, nutrition, chemicals), helping organisms to adapt to adversity [38]. We report that the expression of the C2H2 transcription factor decreases in both the OT-72h and TT-72h treatments (Figure 5B). For HB transcription factors, we report that the expression levels of ct, PRRX1, ZFHX3, Awh, INV, Tgif2, Barhl2, and ey are up-regulated in the TT-72h treatment compared with the TC-72h treatment (Figure 5C). The expression of ABD-A was up-regulated in the TT-96 treatment compared with other treatments. For TRAF transcription factors, most were down-regulated in the OT-72h treatment compared with the OC-72h treatment, while CFDP2, BTBD7, lola, ABTB2, fru, and BTBD8 were down-regulated in the TT-96h treatment compared with other treatments (Figure 5D). TRAFs act as signal transduction proteins and are critical in many biological processes [39,40]. MdTRAF6 is involved in immune regulation and ovarian development, and its absence can lead to higher mortality, lower fertility, and reduced survival of offspring in *Musca domestica* [41]. Changes in expression of C2H2, HB-other, and TRAF transcription factors may affect the reproductive response of silkworm larvae to chlorfenapyr exposure.

PPI results revealed the top 20 proteins associated with reproductive development in ovary tissues (Figure 6A) and 20 ribosomal proteins in testis tissues (Figure 6B). The ecdysone receptor (*EcR*) plays an important role in ecdysteroid signaling, which regulates insect growth, development, and molting [42,43]. Pyruvate dehydrogenase kinase (*Pdk*) is essential for energy metabolism because it modulates pyruvate dehydrogenase activity and influences energy generation in cells [44]. Krüppel homolog1 (*Kr-h1*) is an important effector that mediates the actions of JH and 20-hydroxyecdysone (20E) hormones and promotes vitellogenesis and ovary development in many insects [45]. Triose phosphate isomerase (*Tpi*) is involved in glycolysis and energy metabolism and affects the energy balance of cells [46]. Lactate dehydrogenase (*LDH*) plays an important role in lactic acid fermentation and energy metabolism, regulating energy production in cells [47]. The Forkhead box (*FoxO*) transcription factor regulates insect growth and development by modulating JH degradation [48], reducing oxidative stress, and extending the silkworm lifespan [49]. Glycogen phosphorylase (*Glyp*) is involved in glycogen decomposition and synthesis, and it influences energy metabolism [10]. Silkworm immunity (*Siwi*) is associated with the silkworm immune response and plays a role in pathogen defense [50], and its absence can lead to delayed larval growth and defects in wing development and sexual differentiation [51]. Ribosomal protein S29 (*RpS29*) is involved in protein synthesis and affects cell growth and metabolism [52]. The insect-specific transcription factor Broad-Complex (*BR-C*) is transcriptionally activated by the steroid 20-hydroxyecdysone (20E) and regulates the expression of many target genes involved in insect growth and development [53]. Fushi tarazu factor 1 (*FTZ-F1*) is a class of transcription factors belonging to the nuclear receptor superfamily, which are essential for insect reproduction and the molting process [54,55,56]. In the testis, the main genes identified were members of the ribosomal protein family. Ribosomal protein genes are important in silkworm growth, development, and metabolism, and because of their involvement in protein synthesis, they are essential for cell function and overall organismal health [57]. In summary, these proteins play key roles in silkworm growth, development, and reproduction. Their functions are interconnected, and they form a complex network that regulates insect physiological processes. Future studies could explore the specific roles of these genes in chlorfenapyr-induced reproductive damage and how to mitigate the negative effects of pesticides by regulating the expression of these genes.

Our small sample size increases the risk of false positives (incorrectly identified DEGs) and false negatives (failure to identify true DEGs), which may affect the generalizability and reliability of results. Additionally, while regulatory networks are constructed, we have not experimentally validated these to confirm the role of key transcription factors and their mechanisms of operation. The role of key genes identified in transcriptome analysis could be identified through gene knockout or overexpression experiments.

## 5. Conclusions

We report abnormal development of larval silkworm reproductive tissues following exposure to chlorfenapyr. Chlorfenapyr mainly affects drug metabolism and insect hormone biosynthesis pathways. C2H2, HB-other, and TRAF may be important transcription factors. Expression of a series of genes changed following exposure to chlorfenapyr, which suggests that they might play important roles in gonad development in larval silkworm. We describe a molecular mechanism by which chlorfenapyr affects the reproductive system of larval silkworms, particularly at the transcriptome level, and provide a new molecular perspective for understanding its reproductive development. In doing so, we establish a scientific basis for future ecological safety assessments that involves the application of this pesticide.

## Data Availability

The original contributions presented in this study are included in the article/Supplementary Material. Further inquiries can be directed to the corresponding authors.

## Supplementary Materials

The following supporting information can be downloaded at https://www.mdpi.com/article/10.3390/insects16060619/s1, Table S1: Major primer sequences for qPCR in this study; Table S2: Statistical analyses of RNA sequencing data; Table S3: The DEGs were identified in silkworm larval ovary tissues after exposure to chlorfenapyr by RNA-seq; Table S4: The DEGs were identified in silkworm larval testis tissues after exposure to chlorfenapyr by RNA-seq; Table S5: The transcription factors were identified in silkworm larval gonads after exposure to chlorfenapyr.

## Abbreviations

The following abbreviations are used in this manuscript:

DEGs	Differentially expressed genes
HE	Hematoxylin–eosin
KEGG	Kyoto Encyclopedia of Genes and Genomes
GO	Gene ontology
qPCR	Quantitative real-time polymerase chain reaction
PPI	Protein–protein interaction
PCA	Principal component analysis
HCA	Hierarchical cluster analysis
MCC	Maximal clique centrality
JH	Juvenile hormone
JHAMT	Juvenile hormone acid methyltransferase
JHEH	Juvenile hormone epoxide hydrolase
CYP	Cytochrome P450
GST	Glutathione S-transferase
